# Detection of antibodies against avian influenza in European dairy cattle, the Netherlands, January 2026

**DOI:** 10.2807/1560-7917.ES.2026.31.25.2600464

**Published:** 2026-06-25

**Authors:** Monika Z Ballmann, Luca Bordes, Kim M Bouwman, Marc Y Engelsma, Sandra Venema-Kemper, Sylvia BE Pritz-Verschuren, Rene Heutink, Marit Roose, Marcel AH Spierenburg, Arco van der Spek, Hendrik IJ Roest, Evelien A Germeraad

**Affiliations:** 1 Wageningen Bioveterinary Research, Lelystad, the Netherlands; 2 Incident and Crisis Centre for Animal Diseases and Zoonoses, Netherlands Food and Consumer Product Safety Authority, Utrecht, the Netherlands; 3 Directorate Animal Supply Chain and Animal Welfare, Ministry of Agriculture, Fisheries, Food Security and Nature, The Hague, the Netherlands

**Keywords:** Avian influenza, Highly pathogenic, Cattle, Antibodies, Europe, Field case

## Abstract

In December 2025, highly pathogenic avian influenza (HPAI) H5N1 clade 2.3.4.4b genotype DI.2.1 virus was detected in a cat living on a dairy cattle farm. Milk and serum samples from the dairy cattle were tested for avian influenza virus. No viral RNA was detected; however, H5N1-specific antibodies were identified in serum samples from 34 (47.2%) of 72 lactating dairy cows and 24 (63.2%) of 38 youngstock. These demonstrate expansion of the mammalian host range of HPAI H5N1 in Europe.

Since March 2024, a large-scale outbreak of highly pathogenic avian influenza (HPAI) H5N1 virus has affected dairy cattle in the United States (US) [1]. In contrast, no evidence of HPAI H5N1 infection in dairy cattle associated with the European genotype DI.2.1 has been reported up to date. Here we describe a case investigation and a farm-level survey that resulted in the first reported detection of antibodies against HPAI H5N1 in dairy cattle in Europe.

## Case description and virological investigation of the cat

**Figure 1:**
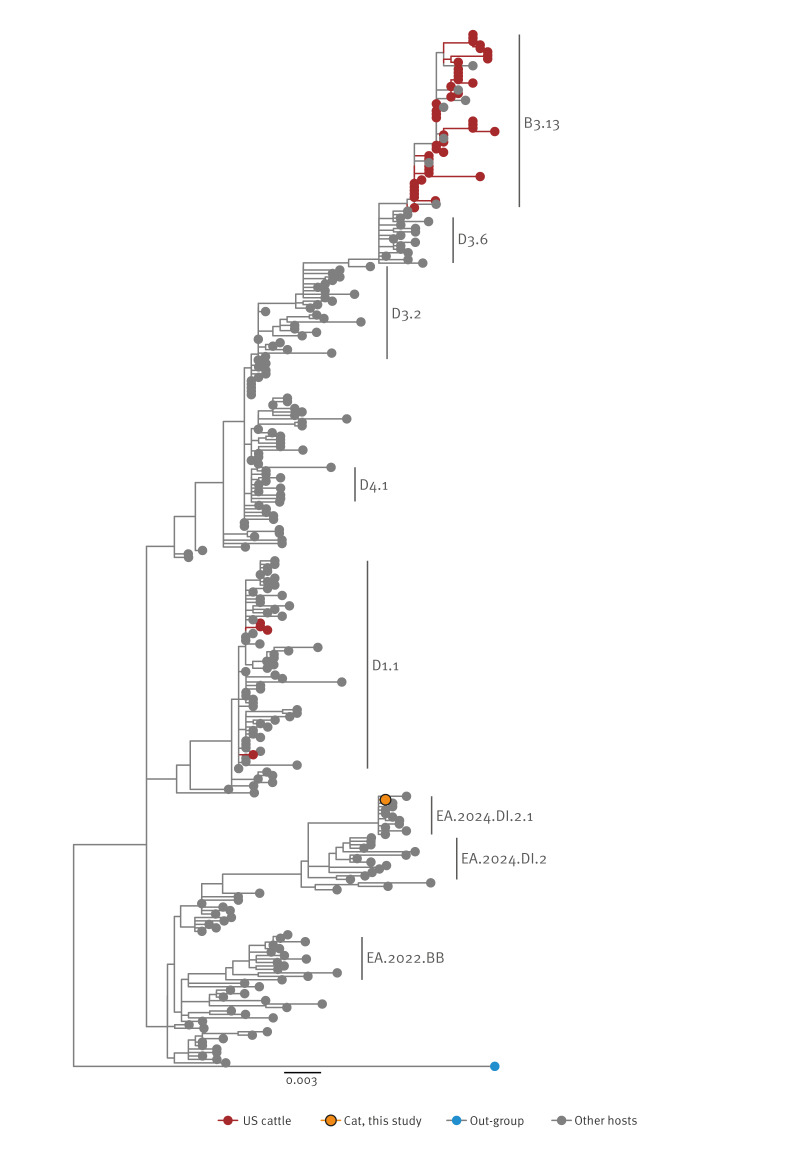
Maximum likelihood phylogenies of the avian influenza A virus H5N1 clade 2.3.4.4b HA genome segment, EU and US sequences, October 2020–January 2026 (n = 297)

EU: European Union; US: United States.

On 24 December 2025, nasal, rectal and conjunctival swabs were collected and submitted for diagnostic testing from a 4-month-old domestic cat living on a dairy cattle farm and exhibiting clinical signs, lethargic and respiratory signs, consistent with HPAI virus infection. The cat died 2 days later. All three swab samples tested positive for influenza A virus by RT-qPCR [2], and the virus was identified as HPAI H5N1 clade 2.3.4.4b genotype DI.2.1 using Illumina whole genome sequencing [3]. Phylogenetic analysis demonstrated that the virus clustered with HPAI H5N1 viruses associated with outbreaks among wild and domestic birds across Europe since October 2025 (Figure 1).

The tree was created using a random set of 1% of the clade 2.3.4.4b sequences shared via GISAID (accession date 27 Jan 2026; https://epicov.org/epi3/epi_set/260216rc?main=true) and the cat sequence (EPI_ISL_20304129) obtained in this study. The strain A/Baikal_teal/Korea/H52/2014 was used as out-group. Other hosts include domestic and wild birds and mammals. Sequences were aligned with MAFFT (https://mafft.cbrc.jp/alignment/server/index.html), phylogenies interfered using IQ-TREE (https://iqtree.github.io/) and visualised with the R package ggtree (https://www.bioconductor.org/packages/release/bioc/html/ggtree.html). Genotype names of GenoFLU and Genin2 are indicated.

The HPAI H5N1 virus detected in the cat did not contain the PB2 M631L substitution that has been frequently reported in the B3.13 genotype in the US, infecting dairy cattle. The PB2 E627K substitution, a well-characterised marker of mammalian adaptation, was present. Furthermore, the PB2 I292V substitution, which was rarely observed in earlier European HPAI H5N1 viruses, but now predominantly identified in the DI genotypes, was detected. No other previously identified genetic markers known to influence virulence, host specificity or binding of host proteins were identified in the cat sequence.

## Investigation of dairy cattle and milk

Following the detection of HPAI H5N1 virus in the cat, dairy cattle from the same farm were tested for avian influenza viruses and antibodies as part of an epidemiological investigation. On 15 January 2026, 20 individual milk samples (midstream milk from all four udder quarters in a single tube) and a bulk tank milk sample were collected. No viral RNA was detected in any of the milk samples by RT-qPCR targeting the matrix gene (M-PCR) [2]. However, antibodies against influenza A virus were detected in nine individual milk samples and the bulk tank milk sample with nucleoprotein (NP)-ELISA (ID Screen Influenza A Antibody Competition Multispecies, Innovative Diagnostics (ID), Grabels, France).

The farm was revisited on 22 January 2026: individual milk (n = 70) and serum (n = 72) samples were obtained from all lactating dairy cows and serum samples from the 38 youngstock (aged 1–2 years) (Table). In this farm-level investigation, all milk and serum samples were tested with NP-ELISA, H5-ELISA (ID Screen Influenza H5 Indirect ELISA, ID) and the haemagglutination inhibition (HI) assay against two H5 antigens, LPAI H5N1 A/Chicken/NL-Swifterband/14002541/2014(EPI_ISL_176754) and HPAI H5N8 A/chicken/Netherlands/20016597-026030/2020 (EPI_ISL_603132) in accordance with the World Organisation for Animal Health (WOAH) Terrestrial Manual [4], using eight haemagglutination units. All samples with antibodies detected in NP-ELISA (except for one milk sample, for which insufficient material was available) were subsequently tested by virus neutralisation test (VNT) using Madin-Darby Canine Kidney cells and a clade 2.3.4.4b H5N1 virus (H5N1 A/Chicken/Netherlands/21037287-006010/2021, EPI_ISL_5588100). A bovine serum sample (A/turkey/Italy/21VIR9520-3/2021 H5N1 subtype, BPL-inactivated HPAI virus, a highly concentrated antigen produced by Istituto Zooprofilattico Sperimentale delle Venezie) containing antibodies against H5 clade 2.3.4.4b, provided by the European Union Reference Laboratory for Avian Influenza, served as the positive control. The test results are summarised in Table.

**Table:** Diagnostic assay results from an investigation of highly pathogenic avian influenza A virus on a dairy cattle farm, the Netherlands, 22 January 2026

Testing	Lactating dairy cows						Youngstock		
	Milk			Serum			Serum		
	Pos. (n)	Tested (n)	%	Pos. (n)	Tested (n)	%	Pos. (n)	Tested (n)	%
NP-ELISA	16^a^	70^b^	22.9	36	72	50.0	25	38	65.8
VNT	14^a^	15^c^	93.3	34	36	94.4	24	25	96.0
H5-ELISA	4	70	5.7	17	72	23.6	9	38	23.7
HI assay^d^	0	70	0.0	11	72	15.3	3	38	7.9
Antibodies^e^	14	70	20.0	34	72	47.2	24	38	63.2

HI: haemagglutination inhibition; NP: nucleoprotein; VNT: virus neutralisation test.

^a^ Bulk milk positive.

^b^ Milk samples were collected from 70 of 72 cows.

^c^ One NP-ELISA positive milk sample could not be tested by VNT due to insufficient sample volume.

^d^ Strains used were H5N1 A/Chicken/NL-Swifterband/14002541/2014 (EPI_ISL_176754) and HPAI H5N8 A/chicken/Netherlands/20016597-026030/2020 (EPI_ISL_603132).

^e^ Samples tested positive in both NP-ELISA and VNT were considered positive for exposure.

**Figure 2:**
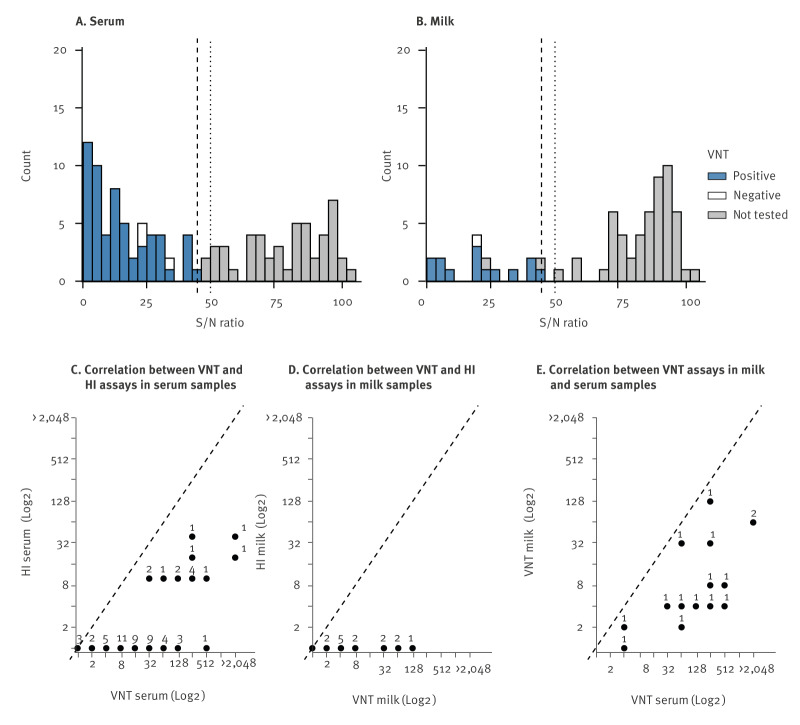
Results of testing serum and milk samples from dairy cattle with nucleoprotein ELISA, virus neutralisation and haemagglutination assay, the Netherlands, 2026^a^

HI: haemagglutination inhibition; NP: nucleoprotein; VNT: virus neutralisation test.

^a^ Number of samples included: Panels A and B: serum (n = 110), milk (n = 70). Panels C–E: samples also tested by VNT: (serum n = 61, milk = 15).

A sample was considered positive if both the NP-ELISA and the VNT showed positive results. Virus neutralisation test results confirmed nearly all NP-ELISA positive results (Table, Figure 2). No HI titres were detected in any of the milk samples, whereas HI titres were measured in 11 (15.3%) of 72 serum samples from lactating dairy cows and 3 of 38 serum samples from youngstock. The HI titres ranged from 10 to 40. In the eight serum samples with the highest VNT titres, antibodies against both H5 and N1 were identified using the multiplex serological assay based on Luminex technology using ProteinAG conjugate [5]. In conclusion, antibodies against H5N1 were detected in serum samples from 34 (47.2%) of 72 lactating dairy cows and 24 of 38 youngstock. Antibodies were detected in milk samples from 14 (20.0%) of 70 dairy cows.

The cut-off of the NP-ELISA for positive samples was 45% (dashed line) and the cut-off for doubtful samples was 50% (dotted line).

The farmer noticed clinical mastitis in one of the lactating dairy cows in December 2025. No other clinical signs were observed by the farmer.

## Discussion

Since March 2024, a large-scale outbreak of HPAI H5N1 clade 2.3.4.4b viruses belonging to the B3.13 and D1.1 genotypes has been reported in dairy cattle in the US [1]. While Eurasian-lineage HPAI H5N1 clade 2.3.4.4b viruses have infected multiple mammalian species [6], including cattle under experimental conditions [7,8], to our knowledge, we report the first detection of H5N1-specific antibodies in European dairy cattle.

In the US, HPAI virus infection in dairy cattle appeared to be mostly asymptomatic or associated with non-specific clinical signs, mastitis, a rapid decline in milk production, reduced feed intake and rumination, lethargy and mild elevation in body temperature [9]. In our report, one lactating cow had clinical mastitis in all four udder quarters. Although the observed clinical signs were consistent with HPAI virus infection, no samples from the symptomatic animal at the time of clinical manifestation were available for testing for influenza virus; therefore, the involvement of other pathogens cannot be excluded. During the investigation, antibodies were detected in more than 50% of the animals suggesting a predominantly subclinical presentation and highlights that infection in dairy cattle may remain undetected without targeted surveillance. While milk is a sensitive matrix for detecting HPAI virus during acute infection in dairy cattle [10], our results indicate that serum could be more suitable for serological surveillance, as antibody titres were higher in serum (VNT titres > 2,048 and up to 40 by HI) than in milk (VNT titres up to 128 and HI titres 0) from the same individual. This finding is consistent with findings from a previous study indicating VNT titres up to 813 in serum and up to 512 in milk [7]. Antibody waning may occur at different rates in serum and milk over time, which may explain the relatively large difference in VNT and HI titres observed in this investigation, but longitudinal studies assessing antibody waning in both serum and milk are currently lacking.

The exact source and route of virus introduction in the cat and cattle remain unclear. The farm is in an area frequented by overwintering birds that grazed on the same grasslands as the cattle. Increased wild bird mortality was observed in the area during October and November 2025. Bird carcasses were removed from the grassed pastures by the farmer but not submitted for virological testing. Around the last weekend of November 2025, dairy cattle and youngstock were moved indoors for the winter, while the cat continued to have access to the fields. The cat may have acquired the virus through consumption of an infected bird or contaminated milk from infected cattle. Cattle exposure may have occurred via contaminated environment, feed or direct contact with wild birds. The high proportion of dairy cows and youngstock with antibodies suggests either a high level of primary exposure or the possibility of cow-to-cow transmission. Further investigation is required to clarify transmission dynamics.

In this study, oropharyngeal swabs collected from members of the farmer's family tested negative for avian influenza virus. Although, since the onset of the outbreak of HPAI H5N1 virus in cattle in the US, occasional human infections have been reported. To date, no sustained human-to-human transmission has been documented. Current public health assessments consider the risk of clade 2.3.4.4b H5N1 viruses to the general population to be low, with human cases limited to sporadic infections following close contact with infected poultry or dairy cattle [11,12]. Consumption of raw milk or unpasteurised dairy products may also pose a risk of virus exposure [13].

## Conclusion

We report detection of antibodies in Europe against the Eurasian lineage of H5N1 virus in dairy cattle, identified after a cat living on the same farm tested positive. Infection of a mammalian livestock species with frequent human contact warrants continuous attention, as it increases opportunities for virus transmission and subsequent adaptation and underscores the importance of integrated surveillance on the animal-human interface.

## Data Availability

All sequence data have been uploaded to the GISAID platform (https://gisaid.org).

## References

[1] Animal and Plant Health Inspection Service (APHIS). HPAI confirmed cases in livestock. Riverdale: APHIS; 13 Mar 2026. Available from: https://www.aphis.usda.gov/livestock-poultry-disease/avian/avian-influenza/hpai-detections/hpai-confirmed-cases-livestock

[2] Bouwstra RJ Koch G Heutink R Harders F van der Spek A Elbers AR Phylogenetic analysis of highly pathogenic avian influenza A(H5N8) virus outbreak strains provides evidence for four separate introductions and one between-poultry farm transmission in the Netherlands, November 2014. Euro Surveill. 2015;20(26):21174. 10.2807/1560-7917.ES2015.20.26.21174 26159311

[3] Beerens N Heutink R Bergervoet SA Harders F Bossers A Koch G. Multiple reassorted viruses as cause of highly pathogenic avian influenza A(H5N8) virus epidemic, the Netherlands, 2016. Emerg Infect Dis. 2017;23(12):1974-81. 10.3201/eid2312.171062 29148396 PMC5708218

[4] World Organisation for Animal Health (WOAH). Chapter 3.3.4. Avian influenza (including infection with highly pathogenic avian influenza viruses). In: Manual of Diagnostic Tests and Vaccines for Terrestrial Animals. Paris: WOAH; 2021. Available from: https://www.woah.org/en/disease/avian-influenza

[5] Fabri ND Santman-Berends IMGA Roos CAJ van Schaik G Het Lam J Germeraad EA No indication of highly pathogenic avian influenza infections in Dutch cows. JDS Commun. 2025;6(3):394-9. 10.3168/jdsc.2024-0703 40458141 PMC12126829

[6] Vreman S Kik M Germeraad E Heutink R Harders F Spierenburg M Zoonotic mutation of highly pathogenic avian influenza H5N1 virus identified in the brain of multiple wild carnivore species. Pathogens. 2023;12(2):168. 10.3390/pathogens12020168 36839440 PMC9961074

[7] Halwe NJ Cool K Breithaupt A Schön J Trujillo JD Nooruzzaman M H5N1 clade 2.3.4.4b dynamics in experimentally infected calves and cows. Nature. 2025;637(8047):903-12. 10.1038/s41586-024-08063-y 39321846 PMC11754106

[8] Bordes L Gerhards NM Peters S van Oort S Roose M Dresken R H5N1 clade 2.3.4.4b avian influenza viruses replicate in differentiated bovine airway epithelial cells cultured at air-liquid interface. J Gen Virol. 2024;105(6):002007. 10.1099/jgv.0.002007 38922678 PMC11256440

[9] Baker AL Arruda B Palmer MV Boggiatto P Sarlo Davila K Buckley A Dairy cows inoculated with highly pathogenic avian influenza virus H5N1. Nature. 2025;637(8047):913-20. 10.1038/s41586-024-08166-6 39406346 PMC11754099

[10] Caserta LC Frye EA Butt SL Laverack M Nooruzzaman M Covaleda LM Spillover of highly pathogenic avian influenza H5N1 virus to dairy cattle. Nature. 2024;634(8034):669-76. 10.1038/s41586-024-07849-4 39053575 PMC11485258

[11] Centers for Disease Control and Prevention (CDC). Highly pathogenic avian influenza A(H5N1) virus: interim recommendations for prevention, monitoring, and public health investigations. Atlanta: CDC; 26 Dec 2024. Available from: https://www.cdc.gov/bird-flu/prevention/hpai-interim-recommendations.html

[12] European Centre for Disease Prevention and Control (ECDC). Detection of avian flu antibodies in Dutch dairy cow: ECDC risk assessment remains unchanged. Stockholm: ECDC; 27 Jan 2026. Available from: https://www.ecdc.europa.eu/en/publications-data/detection-avian-flu-antibodies-dutch-dairy-cow-ecdc-risk-assessment-remains

[13] Kaiser F Cardenas S Yinda KC Mukesh RK Ochwoto M Gallogly S Highly pathogenic avian influenza A(H5N1) virus stability in irradiated raw milk and wastewater and on surfaces, United States. Emerg Infect Dis. 2025;31(4):833-7. 10.3201/eid3104.241615 40072542 PMC11950256

